# Guidance for Efficient Small Animal Imaging Quality Control

**DOI:** 10.1007/s11307-016-1012-3

**Published:** 2016-10-13

**Authors:** Dustin R. Osborne, Claudia Kuntner, Stuart Berr, David Stout

**Affiliations:** 1 Department of Radiology, University of Tennessee Graduate School of Medicine, 1924 Alcoa Highway, Knoxville, TN 37920 USA; 2 Health & Environment Department, Biomedical Systems, AIT Austrian Institute of Technology GmbH, Seibersdorf, Austria; 3 University of Virginia School of Medicine, Radiology, Charlottesville, VA USA; 4 Regis College, Cambridge, MA USA

**Keywords:** Quality control, Small animal imaging, MicroSPECT, MicroPET, MicroCT, Optical, Small animal MRI

## Abstract

Routine quality control is a critical aspect of properly maintaining high-performance small animal imaging instrumentation. A robust quality control program helps produce more reliable data both for academic purposes and as proof of system performance for contract imaging work. For preclinical imaging laboratories, the combination of costs and available resources often limits their ability to produce efficient and effective quality control programs. This work presents a series of simplified quality control procedures that are accessible to a wide range of preclinical imaging laboratories. Our intent is to provide minimum guidelines for routine quality control that can assist preclinical imaging specialists in setting up an appropriate quality control program for their facility.

## Introduction

Adequate quality control is critical for any laboratory producing imaging results used for publication or for industrial contract projects. Quality control (QC) processes enable the testing of the instrument to validate that it is performing to a desired set of specifications prior to obtaining any key results. QC measures for clinical enterprises have long been implemented with many of the quality assurance tests required before any insurance reimbursement can be received for imaging services [1]. The lack of guidance for preclinical quality control can often be a barrier for imaging laboratories to expand their service offerings to include contract research work as well as potentially limits their ability to produce reliable results [2].

Small animal imaging systems often are designed for high-performance data acquisition to enable adequate imaging of small subjects. Although these platforms are advanced in a number of performance aspects compared to clinical systems, they often lack the consistent and robust QC routines that are implemented for all clinical imaging modalities. The lack of automation and standardization, as well as limited staffing in small animal imaging laboratories, can contribute to the use of minimal quality control processes in preclinical imaging laboratories [3].

It is important to begin any quality control program with a reliable baseline. This baseline will be the gold standard for future comparisons of system performance and is critical for understanding the performance of imaging instruments over time. Baseline performance values should typically be obtained during acceptance testing when the instrument is first installed. If you are beginning a new quality control program, then it is important to execute all performance tests initially to obtain the appropriate baseline for future performance comparison.

In addition to setting an accurate baseline, it is critical to log all measured values so that accurate comparison can be made of performance changes over time. One of the key reasons to perform quality control routinely is to create trends in performance that can potentially be used to predict impending failures or system problems. Records of performance values are also important to have on hand to show validation of system performance when discrepancies arise in experimental results.

Finally, all quality control tests within a facility should be documented and recorded as standard operating procedures (SOPs) to ensure consistency of measurements between operators. The SOPs should be controlled documents with some oversight by local expertise and contain all instructions and information necessary to perform the desired QC tasks. The SOPs should also include performance criteria and action levels for each test performed.

This work outlines a unique set of quality control guidelines for primary small animal imaging modalities. Where most quality control guidelines are designed to measure the most stringent performance measurements, the focus of this work is to provide expert-driven suggestions on quality control standards that are simple to perform yet still provide sufficient performance feedback regarding system performance that will enable informed decision making regarding the status of a given imaging platform. These tests are also designed with the knowledge that many laboratories do not have access to specialized phantoms for testing and generally consist of routine tests that can be performed with items commonly found in many laboratories.

## PRECLINCIAL COMPUTED TOMOGRAPHY (CT) QUALITY CONTROL

### CT Overview

Quality control methods for CT are generally straightforward in nature. Although the performance and design of preclinical CT systems vary across manufacturers, nearly all of the commercially available units acquire data in a step-and-shoot fashion rather than through the use of true helical acquisition which requires the use of slip ring technology [4]. The similarities between various configurations enable a more robust definition of standardized quality control methods for preclinical CT.

#### Phantom Setup

The simplest quality control testing for CT uses a simple water phantom with dimensions similar to that of animals or objects imaged in the instrument. A 50-ml centrifuge tube works well as a potential phantom making sure that air bubbles are at a minimum and away from the view normally used in imaging experiments.

For resolution testing on microCT systems, the most common method uses a commercially available wire phantom with a wire diameter that is, at least, half the expected full width at half maximum (FWHM) of the imaging protocol to be tested. Typically, these wires are approximately 5–10 μm in diameter for *in vivo* microCT platforms when testing maximum resolution performance. A wire phantom can be made in the laboratory by using the appropriate amount of wire from another source making sure that the outer diameter is consistent with the diameter requirements to test the desired FWHM resolution.

Alternatively, or in addition to the standard wire phantom, bar patterns may be purchased commercially that can be used to assess 3D reconstructed image resolution [5]. Bar patterns should be chosen to have a number of line pairs per millimeter (lp/mm) of at least half of the maximum lp/mm resolution of the imaging system. These patterns can help to obtain more robust resolution information by enabling the accurate calculation of the modulation transfer function (MTF) for that microCT system [6]. This measurement provides more robust assessment of resolution and system performance than the simpler wire phantom point spread function (PSF) measurement (Table 1).

**Table 1:** CT QC test overview

Daily	Weekly	Monthly	Annually
Tube warmup	Image noise	CT number uniformity	All described QC tests
Detector uniformity	Water CT number		Registration matrix
			Resolution
			Radiation survey

### Daily Tests

For CT imaging, most of the daily tests can be performed rapidly. Although it is recommended that QC be performed daily for the tests below, this is sometimes not possible in preclinical laboratories due to availability of phantoms or lack of dedicated staffing. At a minimum, the following QC procedures should be performed the day prior to imaging to allow time for repairs if needed.

#### Tube Warmup

*Summary:* This test enables adequate warming of the x-ray tube prior to use.

For each day of operation, the x-ray tube should be appropriately warmed prior to use. Many vendors have an automated or manual routine for this function; however, should there be no such protocol or utility, a manual warmup can be performed by creating a series of protocols that begin at low peak kilovoltage and milliamperes and long exposure settings (2 s or more) and gradually increase voltage and current equally in time over a 15–30-min time frame while decreasing exposure times to standard protocol settings [7].

#### Detector Uniformity

*Summary*: This test measures for any visually apparent detector defects and non-uniformities.

**Fig. 1:**
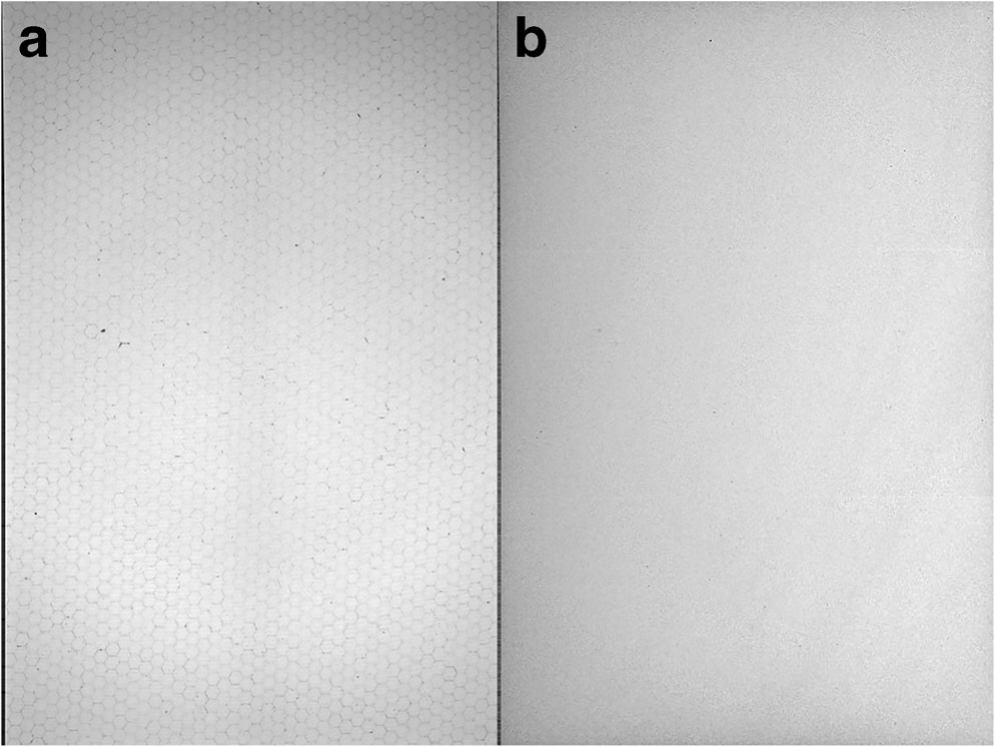
**a** A non-normalized and **b** normalized (*right*) CT projection image taken with no object in the CT FOV using parameters similar to those used by the facility for their specific imaging protocols.

This test is easily performed on a daily basis and requires only a single projection acquisition using protocol settings for your institution’s typical imaging protocol. Uniformity can be assessed using only a visual method for the daily tests. The operator should examine the projection data for any non-uniformities or major pixel defects prior to beginning imaging for the day. Any data collected from the tube warmup procedure can potentially be used to assess uniformity. Fig 1 shows an example of a non-normalized and normalized microCT projection image collected with no object in the FOV.

### Weekly Tests

#### Image Noise and CT Number (Hounsfield Unit)

*Summary*: This test measures whether reconstructed image noise and water CT number are within acceptable values.

For CT systems where the data are used for quantitative measurements or for attenuation and scatter correction in PET and SPECT imaging, assessments of CT image noise and validation of CT number (or Hounsfield Units, if calibrated) are important for maintaining the quantitative nature of measurements. Using the CT protocols used for quantitative results on your system, execute the same scans on the water phantom reconstructed with all settings used for that protocol. Draw a square or cubic region of interest centrally within the phantom with sides of a length at least 7 % the diameter of the phantom used. A minimum of 25 pixels should be included in the region of interest for adequate statistics but should be of a consistent size for each noise test.

The mean should be close to zero. The acceptable range of values for the standard deviation will vary from manufacturer to manufacturer. If the manufacturer does not specify an acceptable range, imaging the phantom multiple times (5–10) and obtaining an average will provide a reasonable baseline for future assessment. Mean values and standard deviation should generally not change by more than 10–20 % [8].

### Monthly Tests

#### CT Number Uniformity

*Summary*: This test assesses the uniformity of a reconstructed water phantom image.

The uniformity of CT numbers should be assessed on a monthly basis. The same guidelines for the regions of interest from the weekly tests should be used; however, in this test, five identical regions should be drawn with one placed centrally within the phantom and the other four placed at the twelve, three, six, and nine o’clock positions. The average variance from the center should be consistent over time and for any given series of measurements should be less than seven.

### Annual Tests

In addition to performing all other QC tests, more stringent assessments should be added and are often warranted on an annual basis.

#### Registration Matrix Generation

*Summary*: This test measures any registration errors that could be apparent between multimodal CT units such as CT/SPECT or CT/PET.

At least once per year and after any servicing of the x-ray tube, x-ray detector, or the gantry the transformation matrices between the CT and other modalities should be repeated. Each manufacturer has a different process for generating these transformation matrices, and those guidelines should be followed in performing these calibrations.

#### Radiation Survey

*Summary*: This test assesses whether the units shielding meets manufacturer and local radiation safety criteria. This test may require Medical Physicist or Radiation Safety Officer Support.

Many microCT systems are self-shielded. On an annual basis, radiation leakage during CT operation should be assessed. Generally, this should be performed by the radiation safety officers for your institutions as they will be aware of the latest guidelines regarding radiation exposure and radiation emitting devices. Any instrument parts or sectors that indicate radiation exposure rates above local guidelines should be repaired by trained service engineers.

### Resolution

*Summary*: This test measures changes in resolution performance over time.

Reconstructed image resolution should be assessed on an annual basis using the wire or bar phantoms. As this resolution test is not to determine maximum resolution but to evaluate resolution performance changes over time, the site should use any protocols commonly used for routine imaging and record the resolution performance for that protocol. This method may also be used to assess maximum resolution performance, if desired, using protocols that enable the maximum magnification, minimum detector binning setting, and reconstruction of data with pixel sizes that are at least half of the expected FWHM resolution for those settings. For maximum resolution assessments, a deconvolution kernel may need to be applied to reach manufacturer specifications.

## PRECLINICAL MAGNETIC RESONANCE IMAGING (MRI) QUALITY CONTROL

### MRI Overview

The quality control methods for preclinical MRI are run to test several components of the system including the magnet, the magnetic field gradients, and the radiofrequency system (send and receive). Quality control for MRI systems tends to be slightly more complex than those for other small animal imaging modalities, requiring specialized phantoms for adequate testing. There are commercially available test phantoms (small and large) designed for clinical MRI that are part of the American College of Radiology (ACR) MRI Accreditation Program [9]. However, no universally agreed-upon phantom exists for small animal MRI QC, but there are some that have been designed and tested for small animal MRIs [10]. One such phantom has been tested using x-ray CT and ultrasound as the gold standard. The construction of this phantom is described here. Computer-aided design (CAD) drawings for this phantom can be downloaded here for 3D printing on a rapid prototyping system.

#### Phantom Setup

The phantom should be filled with a standard solution such as that used in the ACR phantoms, namely, a solution of nickel chloride (10 mM NiCl_2_) and sodium chloride (75 mM NaCl). The nickel shortens the T1 and the sodium chloride provides electrical loading similar to that of a rodent. The orientation of the phantom should be permanently marked on it so as to ensure consistent placement into the MR system. This phantom can be used to ensure the following: (1) the system is geometrically accurate (slice position and thickness), (2) the image intensity is homogeneous, and (3) that there is minimal signal ghosting. The phantom should fit into the RF coils being used, electrically load the RF coil similar to the animals being scanned, have T1 and T2 values similar to those of the tissues being imaged, be easily and reproducibly positioned within the RF coil and the magnet center, and have a region that is free of structures so that a uniformity of signal can be measured.

The phantom tests follow those used for the ACR Small MRI Phantom unless noted otherwise [11].

#### Sagittal Localizer and Protocol Setup

There is no agreed-upon set of standard set of parameters to obtain the MRI QC images for preclinical systems. The following parameter suggestions are based on those used for clinical scanners. Other parameters may be used, but it is extremely important that the same parameters be used each time. Before acquiring these images, a shim and power calibration should be performed (Table 2).

- Sagittal localizer. A 2D gradient echo sequence that covers the entire phantom should be used. Some recommended parameters are TE/TR = 5/15 ms, flip angle (FA) = 20°, readout bandwidth = 130 Hz/pixel, field of view (FOV) = 3 cm, slide thickness = 1 mm, matrix size = 128 × 128, and number of signal averages (NEX) = 1.
- T1-weighted (T1W) axial images. A 2D spin-echo sequence should be used. TE/TR = 650/1500 ms, readout bandwidth = 130 Hz/pixel, field of view (FOV) = 3 cm, slide thickness = 1 mm, matrix size = 128 × 128, and number of signal averages (NEX) = 1. Images should be acquired in three orthogonal planes (sagittal, coronal, and axial) through the center of the phantom.
- T2-weighted axial images. A 2D spin-echo sequence that uses the same slice position and parameters as the T1 W images, except that TE/TR = 75/1000 ms.

**Table 2:** MRI QC test overview

Daily	Weekly	Annually
Magnetic field strength and stability	Geometric accuracy	All described QC tests
Phantom signal to noise ratio (SNR)	Image intensity uniformity	
	% Signal ghosting	

### Daily Tests

#### Magnetic Field Strength and Stability

*Summary*: This test with record keeping is to monitor the primary magnetic field performance over time.

This is determined by finding the resonant frequency of water and keeping a record of this over time. Execution of this test will be specific to each equipment vendor.

#### Phantom Signal to Noise Ratio (SNR)

*Summary*: A test to verify that signal to noise ratio performance meets the appropriate criteria.

An image should be run on a standard phantom, as described above. The image should be acquired daily using exactly the same parameters each time. Two ROIs should be placed in the image, one in the center of the object in the image and one in a place where there is no signal (e.g., air). SNR is calculated with the following formula [12].

$$ SNR=0.655\times \mathrm{mean}- intensity/{SD}_{\mathrm{air}} $$

where mean-intensity is the mean value of the voxels from the ROI drawn in the water region of a phantom and SD_air_ is the standard deviation of the measured voxels from a region that does not contain any water. The correction factor 0.655 is used because noise is normally distributed around zero. Fourier transformation of the raw data and display of a magnitude image (no negative values) result in a skewed distribution of the noise which is corrected by multiplying by 0.655. SNR can be lower due to either a decrease in signal or increase in noise.

### Weekly Tests

Weekly tests are best run at the beginning of the week, especially if the scanner has not been used over the weekend. A single RF coil can be used for these tests and should be the coil that is most commonly used for imaging. However, these tests should be run quarterly on all the RF coils to establish normal values for these coils. If any of the tests fail for the weekly QC runs, another RF coil should be substituted. This will determine whether the tests failed because of the RF coil, which is one of the more common components to fail.

The QC tests described here roughly follow those recommended by the ACR contained in their publication 2015 Magnetic Resonance Imaging Quality Control Manual for clinical imaging systems [9]. These clinical guidelines have been modified appropriately to a set of quality control tests for preclinical scanners to verify the performance of the instruments. The reason for the simplification is to encourage the routine use of these QC tests in every laboratory.

#### Geometric Accuracy

*Summary*: A test to measure the accuracy of the scan geometry and to verify consistency with objects imaged on the system.

This test compares the image-derived dimensions of the phantom with the actual dimensions of the phantom. The exact measurements made depend on the phantom used. The measurements should include the inner diameter (ID) of the phantom from the axial images and the length of the phantom from the sagittal image. The ID should be measured side-to-side, top-to-bottom, and both diagonals. All measurements should be within 2 % of the true value. Inaccurate sizes are likely due to miscalibrated gradients. Each of the primary axes (*x*, *y*, and *z*) has its own calibration factor. Gradients may drift over time. Gradient power amplifiers may need time to warm up before the tests are run. Care should be taken to avoid the use of sequences with a low acquisition bandwidth (long readout time) as this can exacerbate magnetic field inhomogeneities.

#### Image Intensity Uniformity Using the Methods of Firbank et al. [12]

*Summary*: A test to measure the reconstructed image uniformity.

A large ROI is placed in the center of the T1W image. Record the mean, maximum (max), and minimum (min) intensity values. The image uniformity (*U*) is then calculated as

$$ IU=\left( \max - \min \right)/\left( \max + \min \right)\times 100 $$

A number of factors influence the image intensity uniformity. Images become less uniform as the slice position moves away from the center of the RF coil and the magnet. Improper tuning of the RF coil, improper flip angle, or failed RF components can cause image non-uniformity resulting in a decrease in overall SNR. These will also result in a decrease in overall SNR. Uniformity can also be degraded by ghosting which will be tested in the following percent signal ghosting tests.

#### Percent Signal Ghosting

*Summary*: A test to measure displaced signal as a result of ghosting.

Percent signal ghosting is the measure of an artifact that appears as a lower signal replica (ghost) of the object being imaged that is displaced from the main image. The ghost may not appear as a distinct object, but may be smeared signal intensity across the image. This results from some instability that occurs between phase encoding steps, and the ghosts spread out across the image in the phase encode direction. The ghosting is most easy to see in low intensity regions of the image, but also extend through the object being imaged, altering the true intensity.

**Fig. 2:**
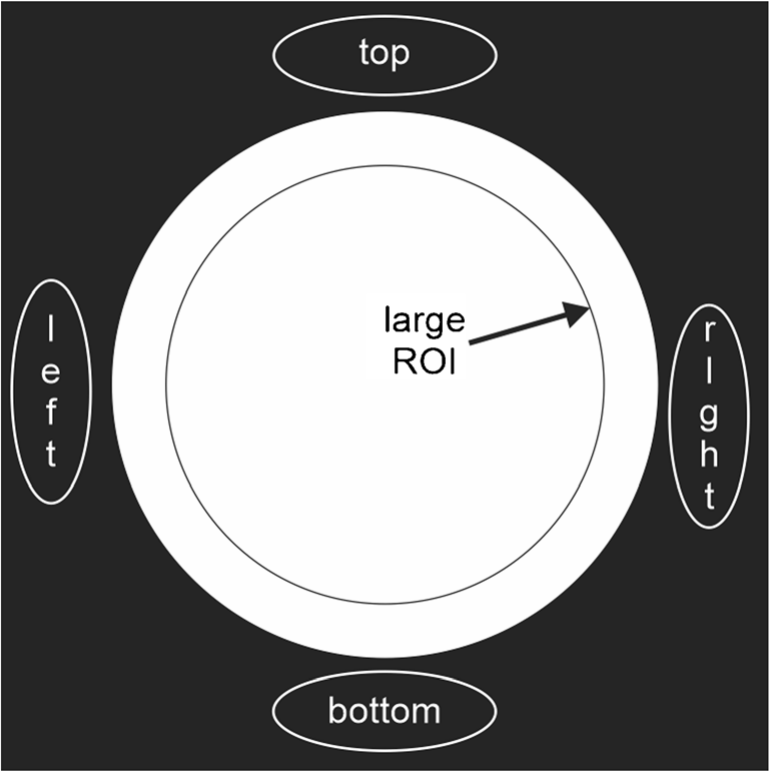
Regions of interest drawn on the typical ACR-like MRI test phantom. This type of coil testing phantom is often a uniform cylinder filled with a 10-mmol nickel chloride solution that appropriately stimulates the coils to have a homogeneous response.

The test image is acquired such that the field of view allows placement of ROIs above, below, and on either side of the object being imaged. Be sure the ROI does not overlap the image or the side of the FOV (Fig. 2). Record the mean of the intensity values for each of the ROIs. The Ghosting Ratio is then calculated as

$$ \mathrm{Ghosting}\;\mathrm{Ratio}=\left[\left(\mathrm{top}+\mathrm{bottom}\right)-\left(\mathrm{left}+\mathrm{right}\right)\right]/\left[2\times \left(\mathrm{large}\ ROI\right)\right] $$

The Ghosting Ratio should be less than 0.025. The most common cause of ghosting is motion of the object being imaged. Be sure the phantom is securely locked down. Other causes of ghosting are due to system instabilities that arise during image acquisition. Tracking this down can be complex and is best left to a service engineer.

## PRECLINICAL POSITRON EMISSION TOMOGRAPHY (PET) QUALITY CONTROL

### PET Overview

Quality control for PET falls into two categories, system setup and routine assessment of performance. System setup involves determining crystal lookup tables, energy settings, and other specifications that are typically done on a one-time basis or whenever system components are replaced or repaired. Performance determinations can be measured using National Electronics Manufacturing Association (NEMA NU 4-2008) standardized methods to obtain image quality and quantitation metrics [13]. Using these standard methods, the performance can be compared to other similar systems and can help with comparisons between different system types.

#### Phantom Setup

**Fig. 3:**
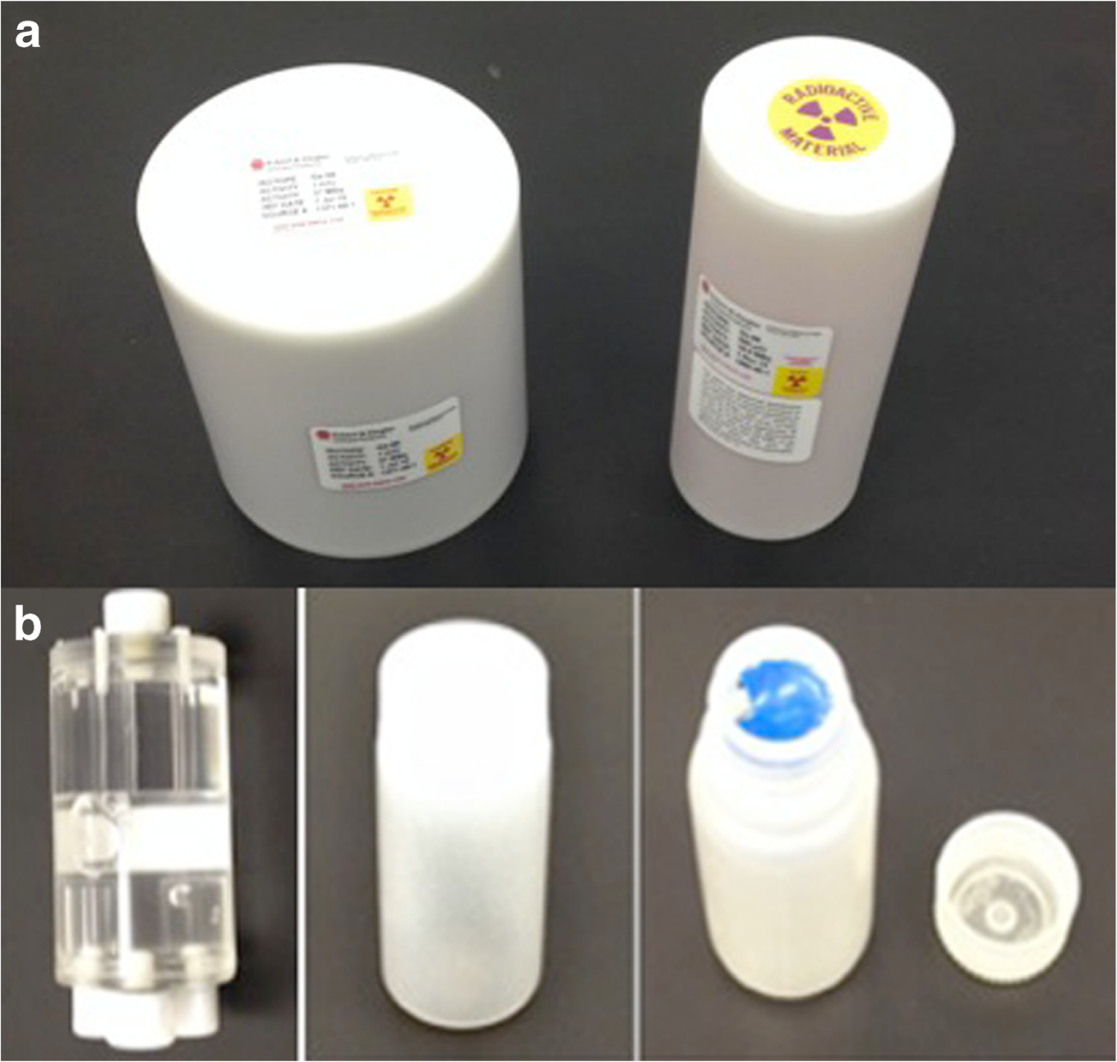
**a** A typical uniform germanium cylinder commonly used for microPET normalization and setup. These phantoms are commercially available from third parties and most vendors. **b** Normalization and setup can also be performed with a fillable 1–2-l bottle making sure the phantom fills the entire axial and transaxial field of view. This phantom was designed by the NEMA NU 4 committee for advanced system acceptance testing and is available commercially from a number of manufacturers.

Routine quality control for PET primarily consists of imaging long-lived isotopes such as Ge-68 or Na-22 encased in epoxy or plastic (Fig. 3a) and imaging of shorter-lived isotopes such as F-18, Cu-64, or I-124 imaged using refillable containers (Fig. 3b). The longer-lived isotopes are good for quick system checks and determining a stable performance over months or years, whereas the short-lived isotopes are useful to cross-validate the gamma counter, dose calibrator, and PET systems with data from imaging experiments. The long-lived phantoms are typically available commercially from the scanner manufacturer and fillable phantom can use uniform cylindrical fillable objects, such as a soda bottles or centrifuge tubes using easily procurable isotopes, such as F-18. For mice, an easy low-cost option readily available is a plastic liquid scintillation vial, which has a volume of roughly 20–28 ml and has a diameter slightly larger than most mice. The vial can be filled with water and activity, with care taken to minimize any air bubble so that the activity is uniformly distributed inside the cylinder (Table 3).

**Table 3:** PET QC test overview

Daily	Monthly	Annually
Visual assessment	PET calibration constant (more frequent if needed)	PET system setup
Efficiency (quick) scan	Gamma counter calibration	Normalization
Dose calibrator constancy		All described QC tests
		Dose calibrator linearity

### PET Daily Tests

#### Visual Assessment and/or Efficiency (Quick) Scan

*Summary*: A basic daily check of PET system function and imaging system performance.

A long-lived source with similar diameter and activity of typical objects imaged in the system is placed in the center of the FOV. A short acquisition of 5–10 min is collected and processed into reconstructed images. The resulting image is then examined visually for artifacts. An ROI containing at least 25 pixels should be drawn and a value for mean and standard deviation calculated. The recorded measurement should not vary from the baseline by more than 20 %.

**Fig. 4:**
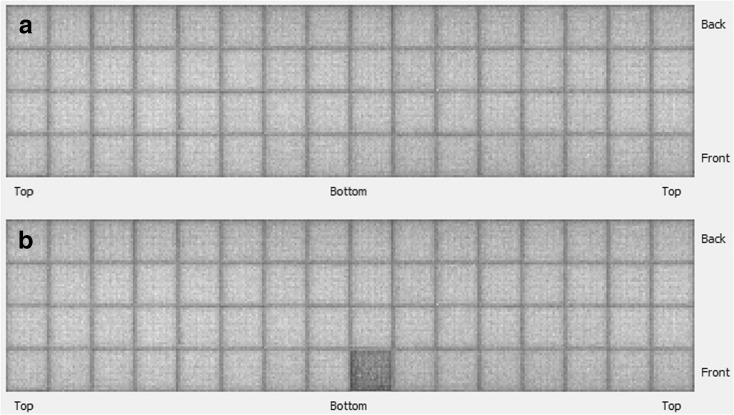
**a** Block efficiency map for a microPET system indicates good uniform performance and **b** a block efficiency map indicating a poor performing detector block. These maps provide information on individual detector block performance.

A visual assessment is not always sufficient to see artifacts, particularly if only a small part of the system fails. The artifact may only be seen in the quantified data or may be hard to see without careful scaling of the image and looking throughout the entire image volume. For this reason, some systems have the ability to look at individual blocks of detectors (Quickscan or efficiency views), which is a quick and easy way to verify that all the electronics are working properly and that each crystal functioning properly. An example of a detector efficiency map (good performance versus poor performance) is shown in Fig. 4a and b.

A long-lived source is recommended for this assessment as it enables long-term tracking of data using a consistent phantom and is better for creating a standard performance baselines as the error in actual activity will be less than that for a hand-filled phantom.

#### Dose Calibrator Constancy

*Summary*: A daily test for the consistency of dose calibrator measurements.

The tests to validate dose calibrator functions are specified by the vendors and are typically both constancy and linearity measurements [14]. For daily or weekly constancy QC, use of a Co-57 or Ge-68 source is a simple, easy measurement to obtain. The values for one or more QC sources can be measured using multiple isotope settings on the calibrator. By measuring the ratio of the isotope settings, if experimental data is inadvertently acquired using the wrong isotope setting, then the measured results can be corrected for the right isotope using the appropriate ratio.

### PET Monthly Tests

#### PET Calibration Constant Testing

*Summary*: A test for the quantitative accuracy of the PET imaging system.

A PET scanner is not used alone as other external accessories, such as a dose measurement device, are required for drawing up doses for injection. Gamma counters are frequently used to measure tissue uptake in *ex vivo* tissue samples following PET imaging. The use of a dose calibrator, PET scanner, and gamma counter to measure radioactivity requires measurement of calibration constants in order to compare results. Calibration constants relate the device measurement to the actual amount of radioactivity, taking into account the sensitivity and performance of the PET scanner. Using these values, the amount of activity drawn in the dose calibrator can be related to the activity in the PET images and to data from blood and tissue samples from the gamma counter.

As implied from the name, this value is expected to be constant, assuming the same settings and methods are used for each measurement. Similar to the daily QC, establishing a baseline and continuing metric for these systems is a useful diagnostic tool in case problems arise. The most common problems arise from using the wrong settings. One example is forgetting to change the isotope setting in the dose calibrator in a location where multiple isotopes are in use. This can happen with the gamma counter, where the wrong energy window, wrong reference decay time, or other wrong settings might be inadvertently used. Likewise, in PET, a wrong protocol might be used that has different settings than desired. When working with short-lived isotopes, the internal clocks on the PET camera PC, gamma-counter PC, and dose calibrator have to be synchronized.

#### PET Calibration

*Summary*: A test to determine the quantitative accuracy of the PET system compared to a paired dose calibrator.

The ideal calibration measurement replicates the conditions for most imaging experiments. This means using the specified activity normally used in routine imaging and a refillable container close to the size and shape of the animal [15]. If the calibration is performed with a lipophilic radiotracer, the solution should be primed with unlabeled compound to avoid adherence of radiotracer to the phantom walls. Acquire a scan similar to normal static imaging times (10–20 min). Because the scatter and attenuation in mice is fairly low, the option to correct for these factors is not essential; however, if the values are going to be quantitatively compared to gamma counter data, then at a minimum attenuation and preferably also scatter correction should be applied during image reconstruction. In the images, a cylindrical region comprising 50–80 % of the radioactivity volume should be consistently applied to obtain the mean region value. By weighing the vial empty and full, and having the dose calibrator measurement of activity used, the activity per unit volume can be determined. Using the ratio of measured activity from the PET scan, and the activity determined from the dose calibrator, the calibration constant value can be determined. Knowing the exact amount of radiation present is difficult due to noise and limitations in each device; however, it is the ratio between these devices which is the primary information required for preclinical experiments.

#### Gamma Counter Calibration

*Summary*: A test to determine the calibration of the PET system to a paired gamma counter.

For gamma counters, the calibration constant is usually called the gamma counter efficiency value. Typical values are 50–65 %, meaning that half to two thirds of the activity in the sample is actually captured and measured. This sensitivity is an order of magnitude greater than most PET systems. Due to deadtime in the electronics processing of events, gamma counters can only measure very small quantities of radiation, typically less than 1 μCi. For this reason, any phantoms or samples measured in the dose calibrator or PET scanner contain far too much activity to assay within a gamma counter. An easy solution is to pair the gamma counter efficiency measurement with the PET scanner calibration constant assay. Using small aliquots of the liquid used in the PET calibration constant phantom, multiple samples can be drawn and assayed to create the gamma counter data. Pipetting small samples is inherently inaccurate, thus samples need to be weighed using an analytical balance to determine the sample volume. From the dose calibrator or PET scanner data, the activity amount in the gamma counter samples can be determined. Ideally, multiple samples are measured to create an average value to avoid noise that comes from human error in handling. Again, when working with lipophilic substances, solutions should be primed with unlabeled compounds. Otherwise, the solution will end up with less radiotracer concentration due to adherence to the walls of the container.

Gamma counters detect radioactivity decay and report counts per second (cps) or counts per minute (cpm). Using the efficiency measurement, these counts can be converted into disintegrations per second (dps), which is the estimated true activity within the detector and is equivalent to 1 Bq of activity.

### PET Semiannual and Annual Tests

#### PET System Setup

*Summary*: This testing requires full setup of the PET system using manufacturer-specified procedures.

Annual performance assessments should begin with a full setup of the PET scanner system using manufacturer-recommended protocols and procedures. Most commonly, these procedures include recalibration of detector high voltage settings, calculation or calibration of pixel location maps, adjustment of electronic calibration factors, and determination of energy lookup tables. Following full setup, the detector system will need to be renormalized as indicated in the following test.

#### PET Normalization

*Summary*: A procedure describing normalization of the PET detector response.

Normalization in PET should be applied to the measured data to level out unavoidable variation in the line of response (LOR) efficiencies due to variations in the crystal efficiencies (due to different light output of the scintillator crystals, different light sharing within the detector blocks, fabrication tolerances, etc.), different positions of the LORs within the FOV, and variations in the photomultiplier tube (PMT) gains. For most of the preclinical PET scanners, normalization is done using the direct normalization method. For this method, a high statistical scan is acquired with a homogeneous source that provides a uniform irradiation of the detectors. These scans usually last 4–6 h and are performed using a Ge-68 cylinder that covers the whole FOV of the scanner. Normalization should be performed for each acquisition protocol on a regular basis (e.g., quarterly or semiannually) or after software or hardware changes. Old or wrong normalization files will lead to artifacts in the images.

#### Dose Calibrator Linearity Testing

*Summary*: A test of the paired dose calibrator linearity performance.

For linearity testing, short-lived isotopes are used in a refillable phantom and placed in a reproducible manner within the detector. Geometry of the source and source volume have a substantial effect on the measurements, so it is important to place the source as close to the bottom of the detector as possible and in the same reproducible way for each measurement. Various isotopes can be used for linearity testing and usually evaluating just one isotope is sufficient. Measurements should be acquired starting with well over the amount of activity typically measured, approximately 3.7 GBq (100+ mCi), depending on the isotope and shipment amounts normally received. Measurements should be made periodically until the source is well below any amounts measured, typically below 0.37 MBq (10 μCi). The data covering the normal range of use is then graphed on semi-log plot, and the results are expected to show a linear loss of activity over time. While this test is often done using C-11 or F-18, it can also be done with longer-lived isotopes if needed.

## PRECLINICAL SINGLE PHOTON EMISSION COMPUTED TOMOGRAPHY (SPECT) QUALITY CONTROL

### SPECT System Overview

Standardization of the quality control methods for preclinical SPECT is a challenging task. Vendors of small animal SPECT imaging equipment use a wide range of collimators, reconstruction methods, and hardware configurations. Adding to these confounding issues, most preclinical SPECT systems also support functionality for planar imaging which, typically, requires a different set of quality control tests. In addition to accounting for differences in instruments from different vendors, each isotope used should be tested independently with initial setup sequences for each isotope including adjusted settings for high voltage, photopeak calibration, and initial uniformity measurements and corrections [16].

#### SPECT Phantom Setup

The phantoms used for SPECT quality control testing are relatively simple. The first phantom required is a point source phantom that can be made using either a partially drawn syringe, capillary tube, or zeolite bead soaked in a solution radioactivity. The typical activity required for preclinical system is on the order of 10–250 μCi. A standardized source helps to maintain better longitudinal records where obtaining daily doses of other compounds may not be feasible in many facilities. If using a non-standard source, then it is advised to use either a commercially available fillable sphere or by soaking zeolite beads for 15–30 min with the appropriate activity concentration to achieve the total activity desired.

For resolution measurements, a line source can be created from a small capillary tube or by using medical tubing taped to rigid cardboard with an inner diameter of at least half that of the expected resolution of protocol or imaging system being tested.

For quantitative assessments, a uniform cylinder with a diameter similar to objects imaged in the system should be used. The phantom should have minimal air bubbles and be filled with up to approximately 250 μCi of activity or to an activity level that produces a dead time of less than 20 % (Table 4).

**Table 4:** SPECT QC test overview

Daily	Weekly	Monthly	Annually
Photopeak drift	Collimator stability	Collimator durability	All described QC tests
Uniformity	Detector stability	Uniformity	Resolution
		Quantitative calibration	Rotational uniformity
			Full SPECT system setup
			Multimodal registration

### SPECT Daily Tests

#### Photopeak Position and Drift

*Summary*: This test monitors the drift of the photopeak for each isotope used for imaging.

This test should be performed without collimators using a point source of 10–250 μCi depending upon the system. The amount of activity used should be enough to acquire the desired counts in a reasonable time frame without exceeding a 20 % deadtime threshold and should ideally maintain a flux of 4500 counts/cm^2^ (not to exceed 10,000 counts per second) [17]. The configuration used should be consistent for daily tracking of photopeak values.

The source should be placed in the center of the FOV with the detector heads moved out as far as possible. For accurate measurements, the distance from the center of the FOV to the detector face needs to be five times the detector FOV. This rule of five times the detector FOV helps to ensure that the detector is being uniformly exposed to the point source but can be a challenging requirement to meet on closed preclinical gamma and SPECT camera gantries [17]. The detector should be appropriately set for the specific isotope prior to beginning data collection.

*Note: If unable to achieve four to five times the detector FOV distance and you obtain unusual uniformity results, you may consider the use of a fillable square/rectangle phantom that covers the entire FOV.*

Use the system to acquire spectrum data using the method described by the manufacturer of the SPECT system. This typically will be either a dedicated panel for monitoring the photopeak in real-time or may require a specific data acquisition protocol to generate the necessary files for analysis. From the data, determine the location of the photopeak which should be labeled in kiloelectron volts. If this value differs by more than 10 % from the initial photopeak setting created during detector setup or baseline, the system should be recalibrated (peaked) per manufacturer instructions. Photopeak drift should be monitored periodically for each isotope used for imaging to verify that the photopeak has not drifted considerably from the desired baseline.

#### Uniformity

*Summary*: This test assesses the uniformity of the SPECT detectors when exposed to a uniform source.

**Fig. 5:**
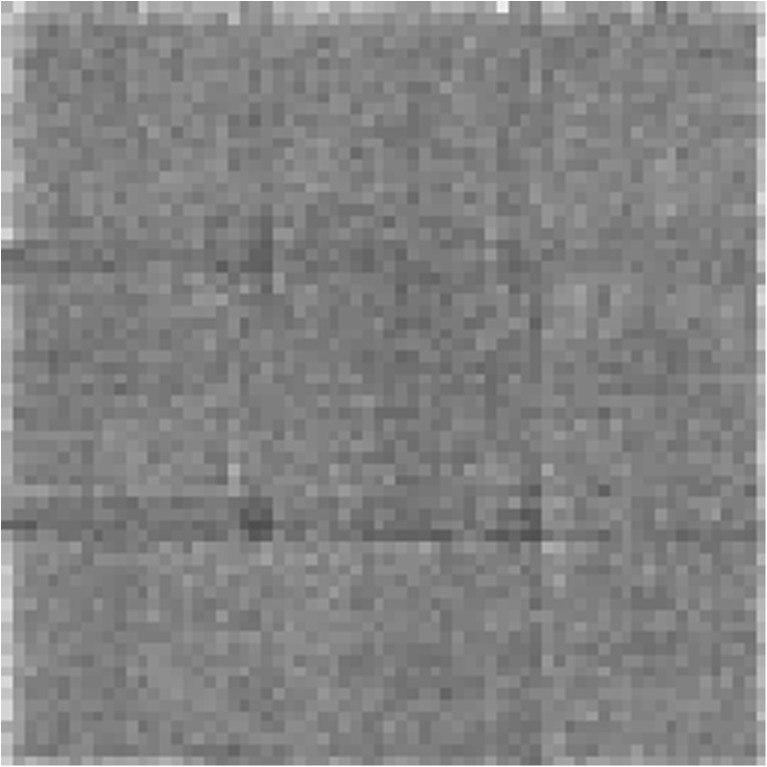
A typical uniform flood image from a microSPECT imaging system generated from a point source phantom located a distance of five times the field of view from the detector system.

Using the same source and five times FOV detector configuration as used in the photopeak drift test, use the system to acquire a planar flood image as shown in Fig. 5. Data should be acquired to achieve a count density of 10,000 counts/pixel for each detector head. For a four-head camera with 50 × 50 pixels, this would equate to collecting 25 million counts per detector (50 × 50 × 10,000).

Visual inspection should be made of the projection data from each detector to assess the image for any irregularities or block detector performance issues. This is the minimum QC that should be performed for uniformity on a daily basis. Often non-uniformities of >10 % can be detected by visual inspection.

Although integral and differential uniformity are recommended by clinical NEMA standards, most preclinical SPECT systems lack the necessary functions to enable easy calculation of differential uniformity. Therefore, it is recommended that, at least, integral uniformity be calculated daily and can be performed using free processing software such as ImageJ or commercially available packages. If your imaging platform includes tools for assessing integral and/or differential uniformity, those should be used for your assessment and both assessments should be performed if possible.

Integral uniformity is calculated by first smoothing the projection images using a standard nine-point smoothing filter [18]. The useful FOV (UFOV) and center FOV (CFOV) are then determined. UFOV is determined by masking the edge of the detector to minimize edge packing effects, while CFOV is determined by taking the central detector area with dimensions (length × width or diameter) 75 % of that calculated for the UFOV. Integral uniformity is then calculated for UFOV and CFOV using the following equation [18]:

$$ UI=\frac{\mathrm{Max}-Min}{\mathrm{Max}+Min}\times 100 $$

Action should be taken if uniformity is found to be greater than 5 % for most preclinical imaging systems.

### SPECT Weekly Tests

#### Collimator and Detector Stability

*Summary*: This test is a physical assessment of the collimators and other scanner components to determine the effects of normal wear and tear.

During the course of routine use, collimator fittings and detector housings can become loosened. This is especially true with preclinical systems that tend to have many different collimator types and may be on automated stages to easily control magnification. These systems also usually have many collimator changes to adjust the system to the appropriate imaging application. Many of these systems are also in enclosed areas where unwanted movement in components cannot easily be seen during acquisition.

On a weekly basis, all collimator components and fittings should be checked that they fit properly. This includes any primary shielding structures or inserts that are available on the imaging system. Detector housings should also be checked to make sure there is no motion when slight pressure is applied. This helps determine that there is no unwanted motion other than the rotation of the detector. If fittings or detector housing couplings to the gantry are found to be loose, the system should be serviced.

### SPECT Monthly Tests

#### Collimator Durability

*Summary*: This test is a physical examination of all collimators used for imaging to check for damage and wear with use.

Preclinical SPECT systems usually have a number of interchangeable collimators that enable a great deal of flexibility but also result in the frequent removal and exchange of these components. This mobility comes at the expense of potentially degrading the integrity of the collimator over time. Each month, imaging staff should check all collimators for signs of physical wear and tear, including dents, fractures, and separation from any framing or structural materials. During this check, collimators should also be attached to the detector head to check that the collimator fits snugly and securely to the attaching mechanism. Loosely fitting collimators can result in significant damage to the SPECT system.

#### Uniformity

*Summary*: Uniformity testing specifications if not possible daily.

If performing integral and/or differential uniformity assessments are not possible on a daily basis, these measurements should be performed, at least, monthly. The same procedures should be followed as indicated in the Daily QC section of this document.

#### Quantitative Calibration

*Summary*: This test is to assess the quantitative accuracy of calibrated measurements.

For SPECT units that support attenuation and scatter correction, a check of the quantitative calibration factor of the system should be performed on a monthly basis for all isotopes. This should be performed using manufacturer-recommended workflows but, at a minimum, should use a cylindrical uniform phantom with an accurately measured activity of approximately 250 μCi (9.3 MBq). The site’s most frequently used protocol can be used for testing but should be set to acquire for approximately 1–2 h to acquire a large number of counts.

Calibration is checked by drawing a cylindrical region of interest in the central part of the phantom and determining the image-based estimation of the activity concentration in the cylinder. The value determined from the reconstructed image of the phantom should then be compared with the actual measured activity in the phantom to determine whether the values are consistent. If these values vary by more than 20 %, the quantitative calibration process for that system or protocol should be repeated. It is imperative to include any data corrections applied to your standard imaging protocol (i.e., scatter and attenuation) when performing the quantitative calibration tests.

### SPECT Annual Tests

#### Resolution

*Summary*: This test is to monitor resolution performance over time. This test is not meant to establish maximum resolution performance or for multiple systems comparison.

At least once a year, resolution measurements should be made to assess the quality of reconstructed images using a standard phantom. Resolution measurements should be performed using a ^99m^Tc line source placed in the center of the field of view. Resolution will be dependent upon the collimator and magnification selected for a given protocol. The goal is not to measure maximum resolution performance but to make sure no significant changes in expected performance are observed over time.

Following acquisition, the data should be reconstructed using the site’s most commonly used reconstruction parameters again making sure they are consistent with previously recorded measurements. A line profile should then be drawn through the hottest central voxel of the point in the tangential and radial directions and the data fit to a Gaussian plus a constant. The FWHM can then be calculated for comparison to previous measurements.

Resolution performance should not vary by more than 10 % or the system should be more stringently assessed for performance issues.

For more stringent testing, this source can also be moved around the active field of view or multiple sources can be created and distributed throughout the field of view to achieve measurements of resolution outside the center of the field of view.

#### Rotational Uniformity

*Summary*: This test assesses detector uniformity at different angles of acquisition.

Because of potential changes in weight distribution, magnetic fields, heat distribution, and mechanical changes as the SP, rotational uniformity should be performed twice per year. Using the same point source arrangement for the daily uniformity assessment, a long, overnight scan covering 360° should be acquired. The raw projection data can then be reviewed for any changes to uniformity as the angle of rotation changes.

#### Full SPECT System Setup

*Summary*: This section describes the recommendations for performing full system setup on an annual basis.

Once per year, the SPECT system should be taken through the entire setup process for all isotopes. This includes full calibration of electronics and detector settings as well as performing new normalizations. For multimodal SPECT/CT units, this should include performing registration and quantitative calibration routines. This time should also be used to perform new geometric calibrations for all collimators.

#### SPECT/CT Registration

*Summary*: This test is to determine any misalignments between multimodal SPECT components.

Over the course of imaging, registration settings can change because of drifts in mechanical aspects of the gantry, including accuracy of bed positioning, and slight movement of components. This test should use either a manufacturer-provided registration phantom or use zeolite beads soaked for 15–30 min in the isotope of your choosing. If using zeolite beads, they should be attached to a cylindrical object and placed asymmetrically around the cylinder. Configure your system for a routine SPECT/CT protocol and then examine the resulting data to determine if there is any mis-registration between the SPECT and CT data. If the difference is visually significant, then rerun the manufacturer SPECT/CT registration calibration routine or call your service provider for assistance.

## PRECLINICAL OPTICAL BIOLUMINESCENCE AND FLUORESCENCE QUALITY CONTROL

### Bioluminescence and Fluorescence Overview

Optical imaging systems are fairly straightforward compared to other imaging modalities with respect to how signals are turned into images. A light-tight box is used in combination with a charge-coupled device (CCD) camera to measure light being emitted from or transmitted through the object being studied. There are a variety of systems available that can acquire simple projection views (2D), multiple 2D views using mirrors, 3D systems where multiple views are obtained using moving mirrors or cameras, and multimodality systems that combine optical imaging with CT, PET, or SPECT.

To see these weak, dim signals, a light-tight enclosure is needed along with a clean interior that does not have any light-emitting substances. CCD cameras have background signals due to several factors; primarily thermal noise related to temperature, cosmic rays, and distortions caused by circular lenses used with square or rectangular cameras. Ideally, the imaging system environment is kept at a stable temperature, and most CCD-based systems have some type of cooling to reduce thermal noise. A flat field correction can be applied to correct for lens distortions, along with cosmic ray correction. The background signal, or dark field signal, is measured and subtracted from each acquired image to correct for variations in the detector pixel signals, normalizing the response in an attempt to provide a uniform flat background response. Often these background measurements are automatically measured on a routine basis late at night.

Obtaining a high-quality, reproducible optical signal requires calibration and verification of system performance on a regular basis. Vendors provide system setup and calibration; however, ongoing verification and testing is required to ensure equipment is working properly and that background subtraction measurements are being measured and properly applied.

#### One Time Setup Checks and Phantom Setup

At the time of initial setup and following any camera repair, the system needs to be (1) calibrated, (2) uniformity of the field of view checked, (3) background acquisition times set up and validated, and (4) filters and software settings checked for proper function. Camera images need to be checked for proper focus, which may involve adjusting the stage height or software settings. Camera resolution can be validated, but this has limited value since all the optical light is scattered in the object in a depth-dependent manner. Settings include default F-stop, binning, and acquisition time. Fluorescent filters need to be validated to make sure they are passing the right wavelengths for excitation and emission filters, and that the software correctly identifies the filter names and locations.

**Fig. 6:**
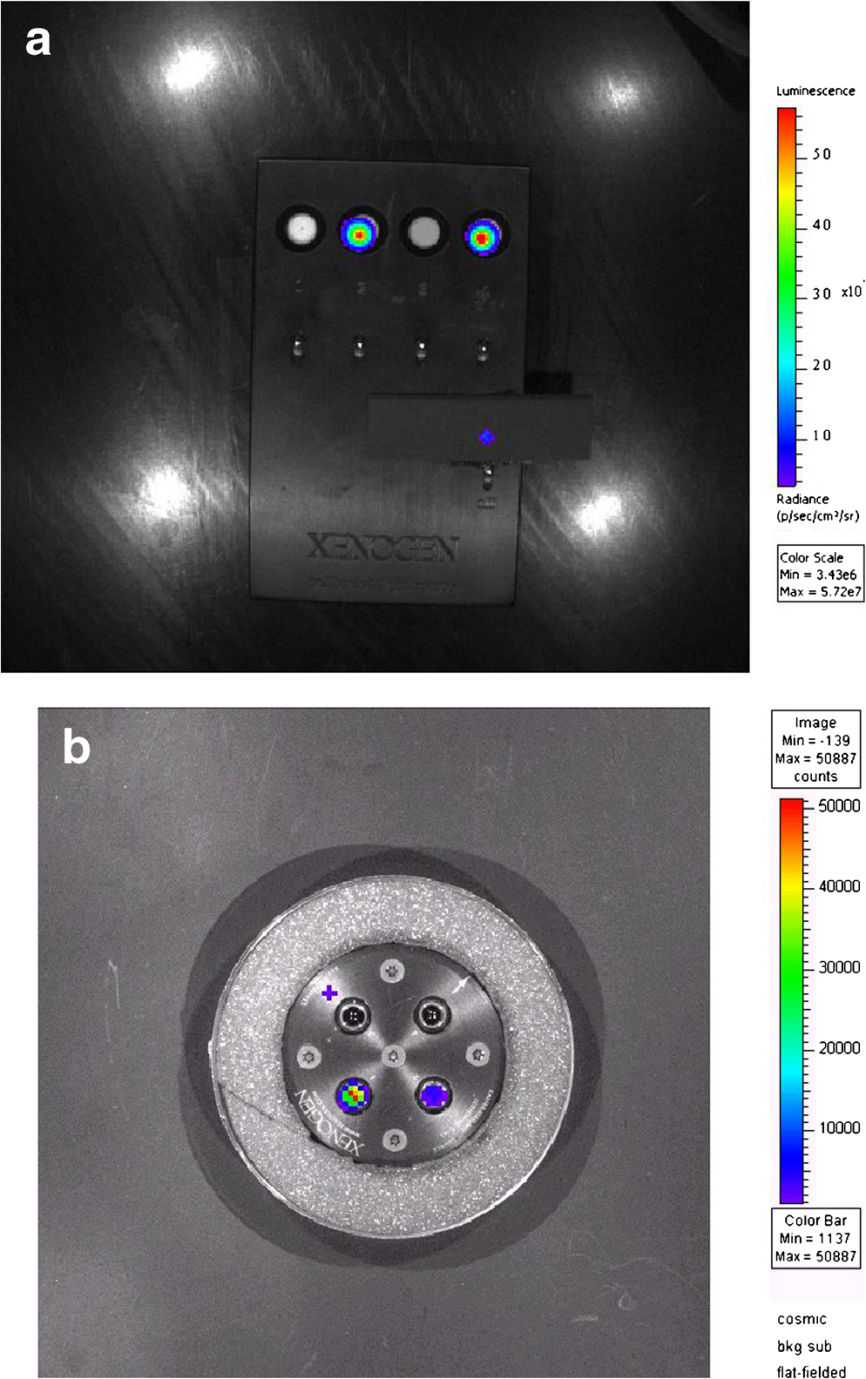
**a**, **b** Two different types of calibrated light sources used in optical system quality control. The light sources may vary between manufacturers; however, all have multiple light sources with accurately known low power emissions. These are imaged with the optical system and used for calibration and QC.

Once these initial startup parameters are validated and in place, there is usually not much need to change these settings. The routine checks are primarily to ensure that the system is continuing to function as determined at the time of acceptance testing using a calibrated light source as shown in Fig. 6. The appropriate light sources can be purchased from third-party vendors and may also be included with the optical products. These settings are often checked as part of an annual service contract maintenance call; however, if desired, they can also be checked by the end users (Table 5).

**Table 5:** Optical QC test overview

Initial	Daily	Monthly	Annually
Full setup	Background visual inspection	Optical performance range	All described QC tests
Camera calibration	Background measurement		
FOV uniformity	Equipment check		
Camera focus			

### Optical Daily Tests

#### Background Measurement and Equipment Check

*Summary*: A basic assessment of the quality of the overnight background correction acquisitions and equipment.

Many optical systems acquire background data at night and create daily files used to correct data acquired throughout the day. Sometimes a problem can arise where these files may not be collected or applied for whatever reason, so a quick check is necessary to see that the files are in place and being properly applied. The most common background measurement errors are due to the door being left open and software or computer inadvertently disabled or turned off. Some optical systems have removable filters or lenses, and if these were not properly put back in place, then the background data may be inaccurate. Check to see that the correct lens and filters are in place and that any associated software settings are correct.

Using a calibrated light source or a reproducible object that provides a suitable signal, a test image should be acquired daily for heavily used systems or prior to any use for less frequently used systems. Check the resulting image for artifacts, that the proper corrections have been applied, and that a region drawn in a known location yields a reproducible signal.

During the daily imaging test, check all the ancillary equipment such as anesthesia and heating systems, imaging chambers, and work surfaces. All should be clean and in good working order.

### Optical Monthly Tests

#### Optical System Performance Range

*Summary*: A test of the system optical performance over a wide range of user protocol parameters.

Once a month, the range of the optical system performance should be measured and checked against previously obtained quality control test data. This can be done using calibrated light sources or a stable imaging phantom such as C-14 radioactivity mixed with liquid scintillation fluid which results in a consistent light source for testing purposes. At a minimum, a wide range of acquisition times should be evaluated, covering the normal range used for experimental conditions. Typically, this will mean acquisitions of less than 1 s up to 3–5 min for bioluminescence. Depending on the system and user preferences, additional tests can be acquired with different settings for binning, F-stop, and stage height.

### Fluorescence Testing

The tests described above will validate the CCD camera performance when performed using a calibrated light source. Unless the fluorescence filters are removed or changed, testing each filter should not be necessary. Validating fluorescence performance using fluorophores is not common, as these samples have diminishing signals over time due to breakdown of the molecules over time and use. Standard kits are available and can be used if desired. These samples can certainly be tested to ensure performance; however, the exact light amount measured will vary due to factors beyond just the system performance, including exposure to room light, storage and temperature conditions, and exposure settings just to name a few.

## DISCUSSION

Although quality control programs can be challenging to implement in the typical preclinical imaging laboratory environment, the benefits to such a program are significant. Firstly, they provide assurances that your equipment is functioning within desired specifications. This increases user confidence of results obtained from imaging equipment and provides solid proof of performance which may be required when contracting imaging services. Secondly, such testing enables timely intervention for issues that require service by providing indications that performance characteristics are drifting from their known quantities. With this series, our goal is to provide preclinical imaging users with basic sets of tests to facilitate implementation of more robust quality control programs.
